# Mental workload classification using convolutional neural networks based on fNIRS-derived prefrontal activity

**DOI:** 10.1186/s12883-023-03504-z

**Published:** 2023-12-15

**Authors:** Jin-Hyuck Park

**Affiliations:** Department of Occupational Therapy, College of Medical Science, Soonchunhyang University, Asan, Republic of Korea

**Keywords:** Classification, Workloads, Functional neuroimaging, Cognitive impairment, Deep learning

## Abstract

**Background:**

Functional near-infrared spectroscopy (fNIRS) is a tool to assess brain activity during cognitive testing. Despite its usefulness, its feasibility in assessing mental workload remains unclear. This study was to investigate the potential use of convolutional neural networks (CNNs) based on functional near-infrared spectroscopy (fNIRS)-derived signals to classify mental workload in individuals with mild cognitive impairment.

**Methods:**

Spatial images by constructing a statistical activation map from the prefrontal activity of 120 subjects with MCI performing three difficulty levels of the N-back task (0, 1, and 2-back) were used for CNNs. The CNNs were evaluated using a *5 and 10*-fold cross-validation method.

**Results:**

As the difficulty level of the N-back task increased, the accuracy decreased and prefrontal activity increased. In addition, there was a significant difference in the accuracy and prefrontal activity across the three levels (*p*’s < 0.05). The accuracy of the CNNs based on fNIRS-derived spatial images evaluated by 5 and 10-fold cross-validation in classifying the difficulty levels ranged from 0.83 to 0.96.

**Conclusion:**

fNIRS could also be a promising tool for measuring mental workload in older adults with MCI despite their cognitive decline. In addition, this study demonstrated the feasibility of the classification performance of the CNNs based on fNIRS-derived signals from the prefrontal cortex.

## Background

It is crucial to understand how the brain allocates mental resources according to cognitive demands for cognitive intervention as an increase in mental workload during challenging cognitive demands can result in poor performance [1]. The concept of mental workload assumes that task-related brain activity consumes a certain amount of mental resources that are proportional to the difficulty of a cognitive task [2]. Therefore, quantifying the amount of energy the brain consumes to meet cognitive demands is one way to measure mental workload.

Many traditional neuroimaging techniques, such as electroencephalography (EEG) and functional magnetic resonance imaging (fMRI), permit the measurement of the neural substrates of mental workload [3, 4]. Although these techniques have enabled an understanding of how the brain interacts with cognitive demands, they are unsuitable for use in ecological contexts. Specifically, subjects must lie motionless and supine during data collection because fMRI is susceptible to a variety of motion-induced artifacts due to head and/or body movements [2]. On the other hand, the advent of wearable EEG devices has notably overcome the limitations associated with immobility. Nevertheless, traditionally EEG measurements were confined to a motionless supine position [5]. Thus, there is a need for ecological measurements that can distinguish between various mental workload levels.

Functional near-infrared spectroscopy (fNIRS) is an emerging and promising imaging technique that satisfies ecological requirements and has the crucial benefit of portability. fNIRS permits *in-vivo* imaging under ecological conditions that allow free movement, in contrast to traditional neuroimaging techniques [6]. This technique has been shown to distinguish between levels of mental workload by measuring the hemoglobin in the blood supply of the brain [6].

To date, fNIRS has been widely used in clinical and aging studies to estimate mental workload [7, 8]. In a related study, consistent changes in oxygenated hemoglobin (HbO2) in the prefrontal cortex (PFC) were found with working memory loads [6]. Another study reported a linear increase in brain activity as working memory loads increased in the PFC [9]. Taken together, increased cognitive demands are coupled with increased prefrontal activity. However, research on this relationship in subjects with cognitive impairment is still lacking despite being fairly well-characterized in healthy subjects [6, 10]. In particular, mild cognitive impairment (MCI) represents a critical stage between normal age-related cognitive decline and more severe conditions like Alzheimer’s disease. Indeed, subjects with MCI consistently showed different neural responses depending on the cognitive load to compensate for cognitive declines based on the PFC’s distinctly impaired function [11, 12]. Consequently, investigating prefrontal activity in this population could offer a unique opportunity to understand early-stage cognitive changes, potentially enabling early intervention strategies.

Therefore, this study was designed to further understand how brain activity is related to mental workload. The purpose of this study was two-fold. Firstly, it aimed to investigate the potential use of fNIRS to estimate mental workload by examining workload-related changes in brain activity in subjects MCI. Secondly, it aimed to construct a classification model using convolutional neural networks (CNNs) for mental workload using fNIRS-derived spatial information on PFC activity during memory testing. Since the classification of spatial information based on fNIRS-derived signals requires an ability to solve high-dimensional pattern problems with a relatively small number of training patterns, conventional classification methods requiring an a priori feature selection process which introduces the possibility of overfitting could not be suitable. However, deep neural networks, in particular CNNs, could bypass the need for feature selection, which has the benefit of enabling learning even in cases where feature selection is not fully optimized [13]. This study hypothesized that increasing the difficulty of a cognitive task would be associated with increased HbO2 in the PFC by compensatory mechanisms to enhance neural recruitment to respond appropriately to increased cognitive demands in older adults with MCI [11, 12]. Therefore, CNNs based on the fNIRS-derived spatial information could differentiate the difficulty levels of a cognitive task.

## Methods

All data were collected from a new cohort and had no overlap with the author’s previous study data. This study measured brain activity in subjects performing a cognitive task using fNIRS, and images from the brain activity of each subject were constructed. The accuracy of CNNs based on the images was analyzed. This study was implemented with the approval of the Institutional Review Board of Soonchunhyang University (202,204-SB-056).

### Participants

The original data set consisted of 120 subjects with MCI. All subjects were older than 65 years of age and recruited from local senior centers in Asan-si, South Korea. Based on an earlier study [10], the inclusion criteria for MCI were as follows: (1) a subjective memory complaint; (2) an objective memory impairment confirmed by performance on neuropsychological assessments (below 1.5 standard deviations); (3) intact global cognitive function confirmed by the Korean version of the Mini-Mental State Examination (MMSE); (4) intact activities of daily living; and (5) without dementia confirmed by a physician. The exclusion criteria were as follows: (1) the presence of psychiatric disorders such as depression or schizophrenia and (2) the presence of neurological disorders such as stroke or traumatic brain injury. These criteria were based on amnestic-MCI, which is a subtype of MCI [14, 15].

This study used the authorized Korean translation of the MMSE supplied by the author’s institution. All subjects completed an informed consent form before participating in this study and all experiments were conducted in the laboratory setting.

### Procedures

fNIRS measured changes in blood flow in the PFC of each subject while performing the computerized N-back task. All subjects were asked to take a rest for 5 min while sitting on a chair in front of the computer monitor and staring at the black cross shape on the white screen of the monitor before the fNIRS measurement. After rest, each subject performed the computerized N-back task, and a keyboard was used as an input device. Blood flow changes in the PFC were measured both at rest and during the N-back task. During the measurement, each subject was instructed not to speak as much as possible and to minimize his/her body movement except for using the input device.

Before the start of the study, all subjects were required to perform the N-back task for practice using the keyboard to adapt to the computerized test. In addition, to ensure clear fNIRS measurements of the PFC areas, all subjects wiped their forehead with alcohol swabs and had their hair trimmed.

### Apparatus

The visual N-back task programmed in Unity was used in this study. This version of the N-back task consisted of three difficulty levels: 0-back, 1-back, and 2-back. Numbers from 0 to 9 were used as stimuli. Stimuli were presented one by one in the center of the screen. On the 0-back task, subjects had to compare the current number to the target number. On the 1-back condition, subjects had to compare the current stimulus to the previous one. On the 2-back condition, subjects had to compare the currently shown number to the one presented two trials before. Each level of the N-back task included 20 trials and was separately conducted.

Subjects were asked to press the “Y” key only when the comparison numbers were the same, otherwise, they were to press the “N” key. A total of 30 target stimuli were presented while 70 non-target stimuli were shown. Each stimulus appeared on the screen for 300ms, followed by a screen that remained blank for another 2,700ms.

The accuracy rate was calculated by dividing the number of correct responses by the total number of target stimuli. To prevent frustration, the task was finished when subjects did not reach a correct percentage of 60%.

To measure hemodynamic responses, the fNIRS device (OctaMon, Artinis, Netherlands) with 8 light sources and 2 light detectors was used at a sampling rate of 10 Hz [16]. The source-detector distance was 30 mm. In this study, only HbO2 which is sensitive to cognitive responses was measured at 760 and 850 nm wavelengths, respectively. According to the international 10–20 EEG placement system, inter-optode distances were set at 3 cm and a total of 8 channels were distributed to target the PFC [17]. All emitters and detectors were mounted on an elastic band to ensure that subjects’ foreheads made good contact with the 10-optode.

### Data preprocessing

All fNIRS data were collected using OxySoft software (version 3.0.52. Artinis Medical Systems BV, Elst, The Netherlands). The concentration changes of HbO2 were computed using the Modified Beer-Lambert Law and then HbO2 data from each hemisphere (4 channels for each hemisphere) were averaged [11, 18]. Channels with large spikes (standard deviation of 300µM/mm) were considered noisy and then were excluded from the analysis [11, 19]. This study used all channels without channel selection as they were all targeting the PFC, which is already widely known to be strongly related to the N-back task. HbO2 data were filtered by a fourth-order Butterworth band-pass filter at a cut-off frequency of 0.1 Hz to remove artifacts such as breathing, blood pressure, and heartbeat [11]. In addition, motion artifacts were removed by a wavelet-based algorithm [20]. Filtered HbO2 data were normalized within the 0–1 range.

### Dataset

**Fig. 1:**
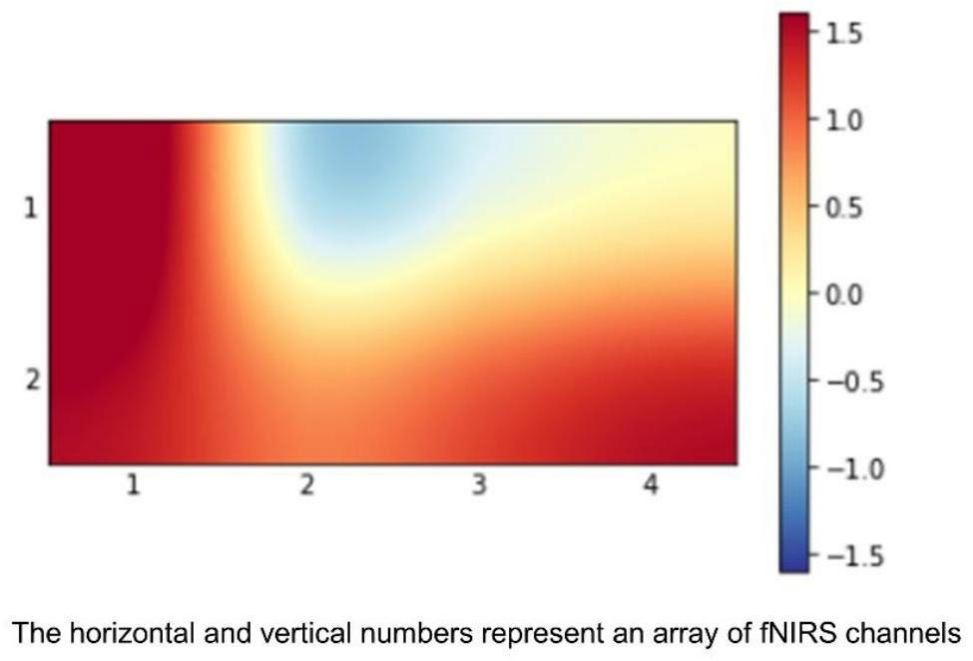
*t*-activation map using oxygenated hemoglobin values from the prefrontal cortex

To use CNNs, the fNIRS signals needed to be converted to data that could be fed into CNNs. Therefore, the averaged time series data were converted to 2D images. 2D representations of the recorded time series based on the spatial coordinates of the 8 channels on the scalp were built. Specifically, the image construction was conducted using a statistical activation map. A statistical *t*-value was obtained to compare the mean of HbO2 from 8 channels between the baseline and the N-back testing periods using a paired *t*-test [21]. The location of each fNIRS channel was assigned to the prefrontal layer according to the international 10–20 EEG placement system and then the *t*-value at each channel was applied to the prefrontal layer (Fig. 1) [22]. The images for 120 subjects were classified into three classes based on the three levels of difficulty of the N-back task including 20 trials, resulting in a total of 7,200 images. The file size of all images was set at 68 × 36.

### CNN model

**Fig. 2:**
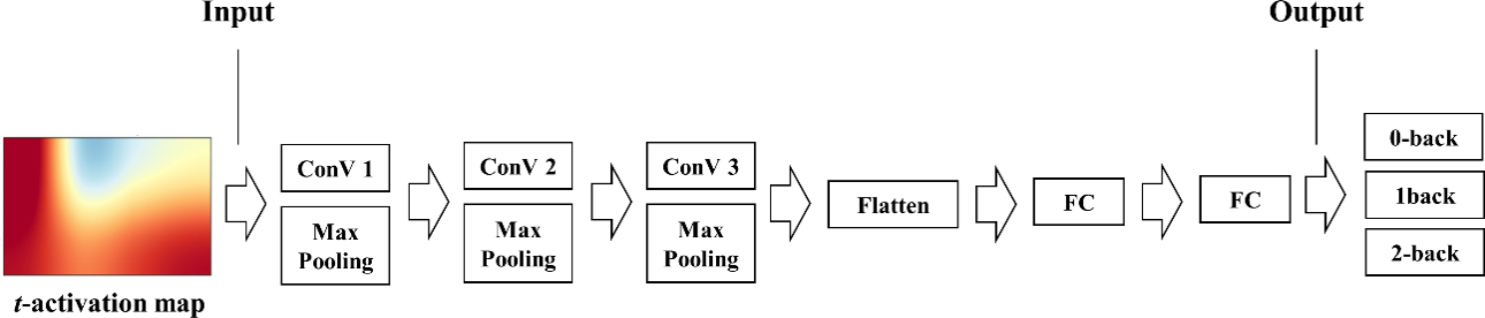
The proposed convolutional neural network model for classifying three levels of mental workload

CNNs were applied to classify mental workloads. The experiment was implemented in Python using the Keras package, with Tensorflow. In this study, CNNs with three convolutional layers including max pooling layers after each convolutional layer and two fully connected layers were adopted. To avoid overfitting, a drop layer was introduced between fully connected layers. The output layer consisted of three units given the dataset (three classes of the N-back task), with a softmax activation. There were 32 convolutions with 3 × 3 kernels with a stride of 1, and zero-padding was applied to maintain spatial dimensions. The two fully connected layers had 256 and 128 neurons, respectively. The dropout rate in the dropout layer was set to 0.5. The rectified linear unit (ReLU) function was used as an activation function (Fig. 2). This CNN model was established according to a previous study [23].

Model training was conducted to increase accuracy and its validation for a maximum of 10 epochs, and the batch size was set to 32. To maximize the validating process, early stopping was arbitrarily applied based on the validation accuracy curve. Categorical cross-entropy was used as a loss function, and the Adam optimizer was used. The ratio of training and test dataset was assigned as 8:2 and *k*-fold cross-validation was used. The data were divided into *k* partitions of equal folds, and CNN training and validation were performed in *k* iterations. For each iteration, one fold was used for testing and *k*-1 folds were utilized in the training phase. This approach systematically partitions the dataset into k subsets, allowing the model to be trained and validated on different portions of the data. By averaging performance across multiple folds, the findings are more reliable, reducing the impact of dataset-specific characteristics (overfitting) and enhancing the generalizability of the findings. Subsequently, the trained model was applied to the test dataset to evaluate the CNN model’s performance, and metrics (accuracy, sensitivity, and specificity) were calculated.

### Neural efficiency

The accuracy across the three difficulty levels of the N-back task and the PFC HbO2 values were each normalized to a range from 0 to 1. Afterward, the neural efficiency index was defined as the value obtained by subtracting the normalized PFC HbO2 values from the normalized accuracy rate by three difficulty levels [2]. The higher the neural efficiency index indicates the higher the neural efficiency.

### Statistical analyses

The accuracy across the three difficulty levels of the N-back task, HbO2 in the PFC, and neural efficiency index were presented using descriptive statistics. A repeated measure analysis of variance was conducted to confirm differences in outcomes by the difficulty levels of the N-back task. Post-hoc was analyzed using multiple paired *t*-tests. To investigate the correlation between the neural efficiency index and accuracy across the difficulty levels, Spearman’s correlation analysis was performed.

## Results

### General characteristics of subjects

Gender, age, education level, and scores of the Korean version of the Montreal Cognitive Assessment (MoCA-K) [24] were investigated. Sixty-two (51.7%) of the subjects were female, and the average age was 74 years. The average education level was 5.5 years, and the average score of the MoCA-K was 22.62 points (Table 1).

**Table 1:** General characteristics of subjects (N = 120)

Characteristics		Subjects
Age (years)		74.97 ± 6.12
Sex	Male	58 (48.3%)
	Female	62 (51.7%)
Education periods (years)		5.55 ± 4.37
MoCA-K (scores)		22.62 ± 1.95

Shown are mean value ± standard deviation. MoCA-K, the Korean version of the Montreal Cognitive Assessment

### Performance on the N-back task and PFC activity

**Fig. 3:**
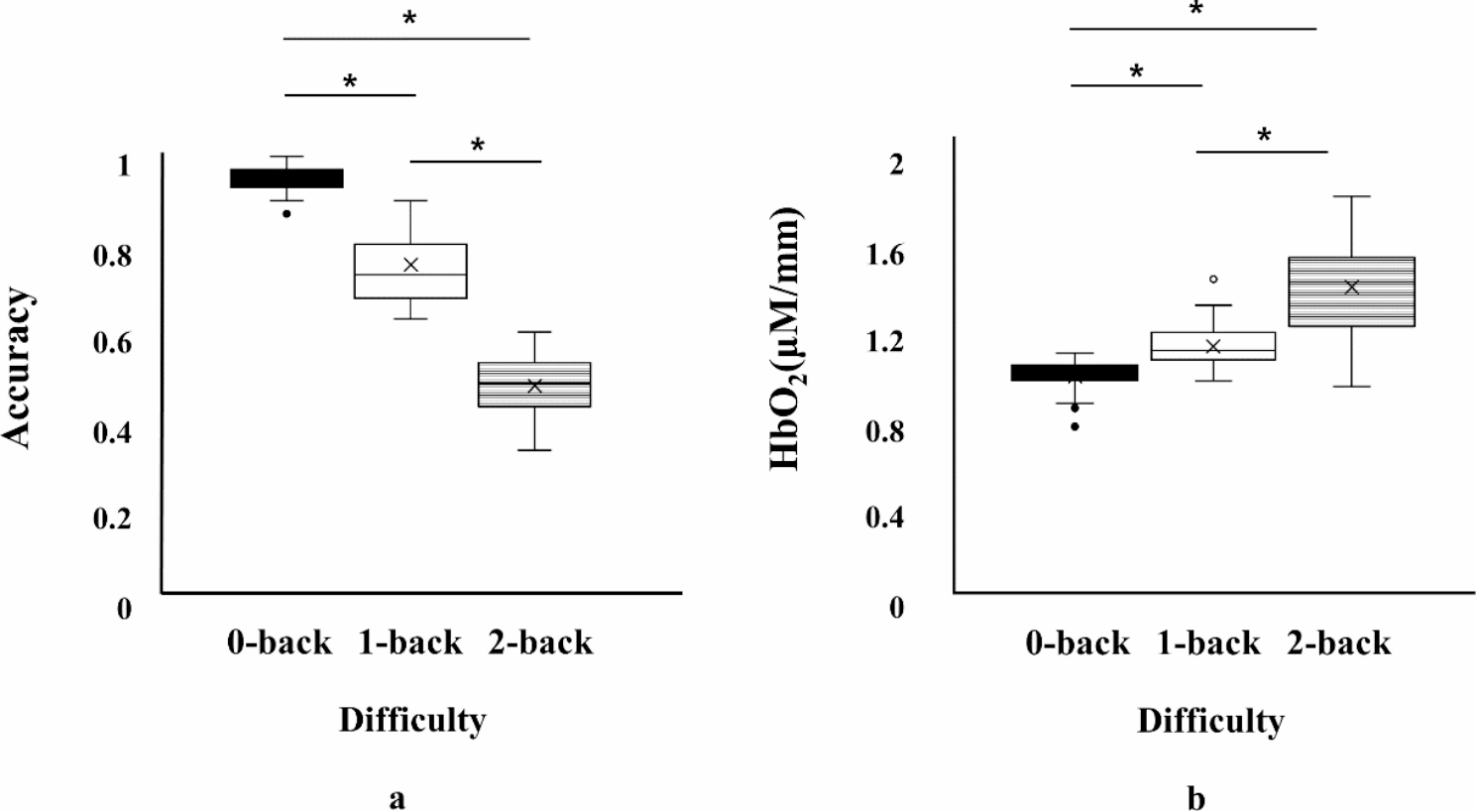
Accuracy and hemodynamic response across the three levels of the N-back task

There was a significant difference in accuracy according to the difficulty levels of the N-back task (*p* < 0.001) (Fig. 3a; Table 2). Specifically, as the difficulty level increased, the accuracy decreased. In addition, there was a significant difference in PFC activity across the difficulty levels of the N-back task. Specifically, as the difficulty level increased, PFC activity increased (*p* < 0.001) (Fig. 3b; Table 2). In sum, increased mental workload induced a decrease in accuracy and an increase in brain activity.

**Table 2:** Accuracy and brain activity across the difficulty levels of the N-back task

0-back		1-back		2-back	
Accuracy	HbO2 (µM/mm)	Accuracy	HbO2 (µM/mm)	Accuracy	HbO2 (µM/mm)
0.952 ± 0.033	0.914 ± 0.072	0.754 ± 0.077	1.040 ± 0.950	0.487 ± 0.068	1.289 ± 0.201

Shown are mean value ± standard deviation. HbO2: Oxygenated Hemoglobin.

### CNN classification accuracy

The accuracy of CNNs across fold numbers ranged from 0.83 to 0.96. *k*-fold cross-validation with k = 10 had the best accuracy, sensitivity, and specificity (Table 3).

**Table 3:** Cross-validation testing for fNIRS-derived spatial images

Approach	Accuracy (%)	Sensitivity (%)	Specificity (%)
5-fold	91.25	91.29	95.62
10-fold	93.33	93.33	96.66

### Neural efficiency index

There was a significant difference in the neural efficiency index across the difficulty levels of the N-back task (F(_2, 78)_ = 7.195, *p* = 0.001) (Table 4). Specifically, the neural efficiency index of the 0-back and 1-back tasks was significantly higher than that of the 2-back task (*p*’s < 0.05). The neural efficiency index of the 0-back task was higher than that of the 1-back task, but there was no significant difference between them (*p* = 0.727). These findings suggested that neural efficiency decreases as the task difficulty level increases. On the other hand, the neural efficiency index and accuracy across the three difficulty levels of the N-back task showed a considerable positive correlation (Table 5), suggesting that the neural efficiency was consistent across the three difficulty levels of the N-back task. Taken together, the neural efficiency index may be a reliable indicator to assess mental workload.

**Table 4:** Neural efficiency index across the difficulty levels of the N-back task

	0-back^a^	1-back^b^	2-back^c^	F	*p*	a,b > c
Neural efficiency index	0.070	0.275	-0.352	7.195	0.001	

Shown are mean value ± standard deviation. HbO2: Oxygenated Hemoglobin

**Table 5:** Correlation between neural efficiency index and accuracy of the N-back task

Characteristics		Neural efficiency index			
		0-back	1-back	2-back	
Accuracy	0-back	0.953^***^	-0.093	-0.015	
	1-back	0.045	0.910^***^	-0.008	
	2-back	-0.078	0.067	0.861^***^	

^***^p < 0.001

## Discussion

This study aimed to validate the feasibility of fNIRS to measure mental workload and to better understand the correlation between PFC activity and cognitive performance. The findings of this study revealed that CNNs based on fNIRS-derived PFC activity during cognitive testing can classify mental workload. Moreover, an increased task difficulty was closely correlated with degraded performances and increased HbO2 in the PFC, resulting in decreases in neural efficiency.

Related studies have consistently reported that oxygenation increases during cognitive tasks such as video games and neuropsychological tests [25–28], which is consistent with the findings of the current study. Notably, despite using only eight fNIRS channels to monitor PFC activity, this study managed to replicate consistent findings from previous studies. A previous study reported that HbO2 concentration in the PFC responds differently according to the difficulty levels of a cognitive task, which further supports the current findings [3]. On the other hand, most previous studies focused on the mental workload of healthy subjects [2, 23, 29], leaving the question of whether fNIRS could be used to measure the mental workload of individuals with cognitive impairment. However, this study measured the mental workload of older adults with MCI using fNIRS-derived spatial information, and the significance of this study was that it confirmed similar findings as healthy people, even though patients with MCI have been identified to show different neural responses to cognitive load compared to healthy people [11, 12]. In other words, fNIRS could be still useful for objectively measuring mental workload in patients with MCI.

The current findings imply that HbO2 concentrations in the PFC from fNIRS can objectively measure mental workload in a laboratory environment. Accordingly, the CNNs based on PFC activity could sensitively classify the difficulty levels of the N-back task. Considering that an indicator based on machine learning techniques could be more sensitive than statistical analysis [15, 30], this study proposed a more optimized index to differentiate mental workload. On the other hand, while prior studies have emphasized temporal information from fNIRS, spatial information analysis with CNN has received less attention [31]. However, in a previous study, CNNs with spatial features showed a considerable improvement in the classification accuracy for mental workload, compared to existing deep neural network methods, supporting the promise of spatial information from fNIRS as proposed by this study [31]. Indeed, in a previous study, CNNs based on fNIRS-derived spatial information achieved 97% accuracy in discriminating mental workload, which supports the findings of this study. However, compared to the findings of this study, the previous study showed higher accuracy, which might be due to the difference in the amount of information collected by fNIRS. The previous study used fNIRS with more channels than this study, measuring not only the PFC area but also the parietal and occipital regions to build CNNs. This disparity suggests the need to observe more brain regions to improve the accuracy of future classification [23]. On the other hand, considering that previous studies that applied traditional machine learning models to fNIRS-derived signals and attempted to classify them for various clinical purposes have reported classification accuracy of less than 85% [32, 33], the high accuracy over 90% of CNNs applied to fNIRS-derived spatial information could support the feasibility of CNNs for fNIRS-derived signals.

On the other hand, monitoring of mental workload by fNIRS could be used for clinical purposes. Real-time monitoring of mental workload could be beneficial to clinicians interacting with subjects performing cognitive tasks, by presenting instant feedback and allowing adjustments to difficulty levels. This would be useful for tailored cognitive training. Indeed, in a prior study, tailored cognitive training using monitoring of mental workload by fNIRS led to a significant improvement in executive function [28]. This customized system could supplement the current difficulty adjustment that depends only on subjects’ performances [3, 34]. Furthermore, in a previous study, fNIRS-derived data could play a role in the diagnostic tool of cognitive disease by providing an objective index [35], supporting its feasibility for a variety of purposes in clinics.

In spite of the clinical usefulness of fNIRS in monitoring mental workload, it is not necessarily associated only with task performance or brain activity [3]. This dissociation could result from individual differences in neural efficiency, where some need more effort for the same output while others require less due to intelligence and expertise [36]. In other words, these differences limit negative correlations between brain activity and cognitive performance. Thus, mental workload with fNIRS needs to be considered in tandem with cognitive performances, as they collectively represent an individual’s neural efficiency. In this study, the neural efficiency index decreased as the task difficulty levels increased, supporting its credibility as a mental workload monitoring indicator.

This study shed new light on the potential use of fNIRS to estimate mental workload. Increased HbO2 in the PFC was associated with increased difficulty levels of the N-back task. Nevertheless, inter-individual variations in intelligence and expertise could limit the correlation between brain activity and mental workload. Thus, mental workload with fNIRS needs to be jointly considered with cognitive performance, which together represent a subject’s neural efficiency.

Although this study shed new light on the potential use of fNIRS to monitor mental workload in people with MCI, there were limitations. One of the main limitations was the lack of consideration for expertise in specific cognitive domains, impacting cognitive performances and neural efficiency in domain-specific cognitive tasks [37]. Therefore, future studies need to compare individuals with different experience levels on cognitive tasks to better understand the effects of practice. The second issue may be the test duration. The N-back task duration was quite long, which could increase variability in hemodynamic responses. Indeed, a prior study indicated that long task durations failed to find correlations between brain activity and cognitive performance [38]. Thirdly, while the results of this study are consistent with those of previous studies in healthy subjects, this study was unable to directly compare the performance of the model in healthy subjects and determine how it differs from the models in older adults with MCI. Therefore, in future studies, a comparative study needs to be conducted. Finally, since this study used all channels without any channel selection techniques, there is still room for further optimization of the accuracy. However, the fact that this study achieved over 90% accuracy without them still supports the methodology of this study using all channels that focally targeted the PFC. Nevertheless, channel selection might need to be used in the future when using fNIRS with a larger number of channels measuring multiple brain regions.

## Conclusions

fNIRS could also be a promising tool for measuring mental workload in older adults with MCI. Despite their cognitive decline, older adults with MCI showed correspondingly higher prefrontal activity as the difficulty of the cognitive task increased. In addition, this study demonstrated the feasibility of the classification performance of the CNNs based on fNIRS-derived signals from the prefrontal cortex. However, brain activity might not be a sensitive indicator of cognitive performance, highlighting the importance of neural efficiency as a more reliable measure.

## Data Availability

The data presented in this study are available on request from the corresponding author.
